# Acceptability and feasibility of the CHARISMA counseling intervention to support women’s use of pre-exposure prophylaxis: results of a pilot study

**DOI:** 10.1186/s12905-021-01262-z

**Published:** 2021-03-25

**Authors:** Ellen K. Wilson, L. Danielle Wagner, Thesla Palanee-Phillips, Sarah T. Roberts, Elizabeth E. Tolley, Florence Mathebula, Laura Pascoe, Michele Lanham, Rose Wilcher, Elizabeth T. Montgomery, the CHARISMA team

**Affiliations:** 1 Women’s Global Health Imperative, RTI International, San Francisco, CA USA; 2 Wits Reproductive Health and HIV Institute, Johannesburg, South Africa; 3 FHI 360, Durham, NC USA; 4 Sonke Gender Justice, Cape Town, South Africa; 5 RTI International, 2150 Shattuck Avenue, Suite 800, Berkeley, CA 94704 USA

**Keywords:** HIV prevention, Microbicides, PrEP, Intimate partner violence, Evaluation, South Africa

## Abstract

**Background:**

Women may need or seek male partner approval to safely and consistently use oral antiretroviral pre-exposure prophylaxis (PrEP) or vaginal microbicides. We developed CHARISMA, a counseling intervention to support women’s relationships and their ability to consistently use HIV prevention products.

**Methods:**

In a pilot study with 95 female participants in Johannesburg, South Africa, lay counselors implemented CHARISMA, assessing participants’ relationship(s) with their male partner(s) and barriers or facilitators to HIV prevention method use, and then providing tailored, interactive counseling. We conducted study participant surveys and clinic staff interviews to evaluate CHARISMA’s feasibility and acceptability.

**Results:**

The CHARISMA pilot study indicates that a two-session relationship counseling intervention with 6-month follow-up to support women’s ability to safely and effectively use vaginal microbicides was generally acceptable and feasible. Most participants thought CHARISMA was relevant, helpful, and about the right length, and that it had a positive impact on their relationships with their partners and their product use. Staff estimated that the intervention took 1.5–2 h to implement at enrollment and 45 min to an hour for the month 1 visit. They thought that overall CHARISMA was generally feasible to implement.

**Conclusions:**

Findings from this study suggest several lessons learned that may be relevant to others developing interventions supporting women’s use of oral PrEP or vaginal microbicides. The use of lay counselors instead of nurses to deliver counseling appeared to be successful, but the counselors experienced significant stress from hearing about participants’ traumatic experiences and required emotional support to avoid burnout. Although staff and participants felt that having multiple intervention sessions over time was valuable, a similar level of intensity may not be feasible in other settings. Further research is needed to determine an intervention delivery mode and follow-up period that optimally balances participant needs and clinic resources. Male engagement was a challenge, as it has been in previous studies of vaginal microbicides. Alternative strategies to reach men that do not require them to come to the clinic or rely on their female partners may be more effective.

**Supplementary Information:**

The online version contains supplementary material available at 10.1186/s12905-021-01262-z.

## Background

HIV prevention products such as oral antiretroviral pre-exposure prophylaxis (PrEP) and vaginal microbicides have the potential to empower women to protect themselves from HIV infection, given that women can use them without a partner’s knowledge, approval or support [1–3]. In practice, however, male partner approval or active support is often desired, required, or culturally indicated for women’s use of these products [4–6], and several studies in southern Africa have found that most study participants (65–87%) disclose their product use to their partners [7–9]. As a result, male partner resistance can pose a significant barrier to the uptake of and adherence to these products [7, 10–12]. Traditional gender norms that position men as the authority in the household compound the impact of partner resistance on women’s ability to use HIV prevention products [13, 14]. Intimate partner violence (IPV) also affects women’s ability to protect themselves from HIV infection, as women in abusive relationships are less likely than non-abused women to refuse sex or use condoms during intercourse [15–18], and some women may experience IPV or other social harms in reaction to their use of HIV prevention products [10, 18, 19].

To support women’s ability to safely and consistently use HIV prevention products, we developed and pilot tested the Community Health Clinic Model for Agency in Relationships and Safer Microbicide Adherence (CHARISMA) intervention. CHARISMA was designed to increase women’s agency to safely and consistently use HIV prevention products, constructively engage male partners in HIV prevention, overcome harmful gender norms, and reduce IPV.

We conducted a pilot study of the CHARISMA intervention with women participating in the Microbicide Trials Network (MTN)-025 HIV Open-Label Prevention Extension (HOPE) study at the Wits Reproductive Health and HIV Institute (Wits RHI) site in Johannesburg, South Africa [20]. The MTN-025/HOPE study, which began in mid-2016, offered women who had participated in the MTN-020/A Study to Prevent Infection with a Ring for Extended Use (ASPIRE) phase III clinical trial of the dapivirine ring an opportunity to continue to use the ring in the context of an open label extension study on safety and adherence. All women participating in HOPE at the Wits RHI site were invited at their enrollment visit to participate in the CHARISMA pilot study (“CHARISMA”).

We sought to evaluate the feasibility and acceptability of CHARISMA as implemented during the pilot study to inform further refinement and testing of the intervention.

## Methods

### Intervention description

CHARISMA was implemented by lay counselors who had previous experience in HIV counseling and testing. For CHARISMA, the counselors received six days of training on gender and violence, sexual relationship dynamics, empowerment counseling skills, and understanding the specific CHARISMA counseling modules and approach. CHARISMA included a core session implemented at enrollment, a shorter booster session at the month 1 HOPE visit, and ongoing follow-up through their month 6 HOPE visit. If participants reported having a new partner at any time during the 6-month follow-up, counselors re-initiated the intervention, and if participants reported experiencing IPV after the core session, counselors provided them with enhanced counseling and referrals, as needed. Because HOPE participants were using the dapivirine ring, the pilot version of CHARISMA was tailored specifically to support ring use, rather than other forms of HIV prevention.

During the core session at enrollment, the lay counselors screened participants using a tool called the HEAlthy Relationship assessment Tool (HEART), which assessed the quality of participants’ relationship(s) with their male partner(s) and potential barriers or facilitators to use of HIV prevention products. The counselors then provided skills-based, interactive counseling based on a modified version of the Safe & Sound IPV prevention intervention [21]. Modifications included adapting Safe & Sound to a non-pregnant population and the use of lay counselors instead of using nurses to implement the intervention. CHARISMA counseling included a brief module on healthy relationships and a module on either partner communication, ring disclosure, or IPV prevention, depending on the participants’ responses to the HEART and the counselor’s assessment of their needs. At the end of the core session, counselors guided participants to develop action plans for how they could apply some of the skills they gained during the session to their lives. Counselors also provided referrals, as needed, to organizations in the community for additional services (e.g., psycho-social, legal, medical). For the booster session at month 1 HOPE visit, CHARISMA counselors followed up on progress around action plans (i.e., whether participants were able to take action and what happened) and offered booster counseling according to the participants’ needs. CHARISMA counselors collected follow-up HEART data collected at participants’ month 3 and month 6 HOPE visits and any time that participants reported a new partner.

CHARISMA also sought to increase men’s awareness, acceptance, and support for women’s use of the dapivirine ring. Women participating in the pilot were encouraged to invite their partners to come to the clinic for either individual or couples counseling because previous studies of vaginal microbicides have found that very few male partners responded to invitations to come to the clinic [5]. However, CHARISMA also conducted outreach to men in the general community at the broader community level. Local project partner Sonke Gender Justice (Sonke) formed community action teams in the two Johannesburg communities where most CHARISMA participants lived (Hillbrow and Diepkloof) to conduct community engagement activities with men; to challenge harmful norms around gender, intimate partner violence, and HIV prevention; and to raise awareness of and support for the vaginal ring. Additional details about CHARISMA have been published elsewhere [22].

### Data collection and analysis

Data are drawn from two sources: a cross-sectional survey of women participating in the clinic component of CHARISMA and key informant interviews (KII) with Wits RHI staff involved with the clinic component of CHARISMA. We also conducted KII with members of Sonke staff and the community action teams working on the community component of CHARISMA, but data from these interviews are not included in the present analysis, which focuses on the feasibility and acceptability of the clinic component of CHARISMA. The survey questionnaire and KII guide developed for the evaluation are provided as Additional file 1.

For the cross-sectional survey, evaluation questionnaires were administered to CHARISMA participants between November 2017 and May 2018 at the Wits RHI clinic by non-CHARISMA staff upon completion of the CHARISMA intervention (i.e., the month 6 study visit) or soon thereafter. Questions focused on participants’ overall thoughts on CHARISMA and feedback on specific aspects of the intervention, including the HEART, the counseling modules they received, the intervention staff, and whether they would prefer administration of CHARISMA by a counselor or self-administration by computer in the future. The questionnaire consisted mostly of closed-ended questions, but also included several open-ended questions.

We conducted KII with CHARISMA clinic staff in June 2017, approximately 6 months after enrollment into the pilot study began. Eight staff members were interviewed, representing all the CHARISMA clinic staff except for 1 counselor who was unavailable. They included 3 counselors, 4 managers, and 1 community engagement liaison. Two members of the CHARISMA monitoring and evaluation team conducted the interviews in person using a semi-structured interview guide. The interview guide addressed five domains which may influence implementation of an intervention, as laid out in the Consolidated Framework for Implementation Research (CFIR): intervention characteristics, inner setting, outer setting, characteristics of individuals, and implementation process [23]. Questions addressed the content of the intervention, how the intervention was implemented, factors that hindered or facilitated implementation in each of the five CFIR domains, staff’s perspective of participant reactions to the intervention, perceptions of the intervention’s effectiveness, and thoughts on how the intervention could be improved. Interviews were conducted in English, audio-recorded and transcribed verbatim.

We analyzed participant questionnaire data by generating frequencies for each of the quantitative questions and conducting content analysis of the responses to open-ended questions. Pseudonyms were used to present quoted responses to open-ended questions. For analysis of the KII data, the evaluation team developed a codebook based on the topic domains for the interim review. Following this codebook, the team used NVivo 11 qualitative data analysis software to code the transcripts. Three staff conducted coding and met regularly to discuss revisions needed to the codebook, coding decisions and intercoder reliability. The team used content analysis to identify core themes and patterns.

Procedures for cross-sectional surveys were approved by the Wits RHI Human Research Ethics Committee for use with the participants who provided written informed consent to participate in CHARISMA. Staff KII were classified as program evaluation activities and not human subjects research by the US-based institutional IRB; consequently, only oral consent was obtained from staff.

## Results

### Participant characteristics

All 95 HOPE participants who enrolled during the CHARISMA enrollment period agreed to participate in CHARISMA; an additional 5 HOPE participants enrolled after the CHARISMA enrollment period had closed and did not participate in CHARISMA. Ninety-two of the 95 CHARISMA participants completed the evaluation questionnaire. At enrollment in CHARISMA, the average age of the respondents was 30 (Table 1), with a range of 21–48. All of the respondents were black, and over two-thirds had at least a secondary school education. Thirty-four percent lived with a partner, 65% did not live with a partner, and only 17% were married. Twelve percent reported that their partner had committed physical or sexual violence against them in the 12 months before enrollment. Fewer than half (45%) earned their own income.

**Table 1:** Participant characteristics at enrollment (n = 92)

	Mean (SD)
Age	30 ± 7
	N (%)
*Race*	
Black	92 (100)
*Highest level of education*	
Primary school	3 (3)
Secondary school, not complete	24 (26)
Secondary school, complete	44 (49)
Any college or university	21 (23)
*Living with primary partner*	
No primary partner	1 (1.1)
Lives with partner	31 (34)
Does not live with partner	60 (65)
*Marital status*	
Currently married	16 (17)
Not currently married	76 (83)
Any physical or sexual violence, past 12 months^a^	11 (12)
Slapped, hit, or beaten by partner	10 (11)
Kicked, dragged, or pushed by partner	7 (8)
Forced to have sex by partner	3 (3)
*Participant earns own income*	
Yes	41 (45)
No	51 (55)
Total	92 (100)

SD = standard deviation

^a^ Aggregate measure that includes report of one or more of the below experiences

### Acceptability of CHARISMA

As shown in Table 2, all 92 participants who completed the questionnaire were administered the HEART tool and received the healthy relationships counseling module. Participants received supplemental counseling modules based on HEART tool recommendations and counselor assessments. Most participants (n = 54) received the IPV module; fewer received the partner communication module (n = 30) or the ring disclosure module (n = 27). Participants generally found the questions in the HEART tool easy to answer (70% very easy and 20% somewhat easy). Only a quarter of participants (25%) found the questions to be highly relevant, but an additional 61% found them somewhat relevant, and a large majority (79%) reported that they were very helpful. When asked why they found the HEART helpful, the primary reason participants gave was that it helped them to understand problems in their relationships and, in some cases, motivated them to make changes. As one participant said, *“[The questions] were helpful because they sort of made me realize the issues I am going through, and I was able to get counseling afterwards.”* (Lindiwe, age 20–24). A secondary reason participants said the HEART was helpful was that it gave them a new perspective on gender roles: *“It helped me in understanding…that men and women are equal and that we need to share in duties in the house.”* (Ndondoloza, age 20–24).

**Table 2:** Participants’ reactions to CHARISMA components

	HEART (n = 92)	Healthy relationships counseling module (n = 92)^a^	Supplemental counseling modules		
			Partner communication (n = 30)^a^	Ring disclosure (n = 27)^a^	IPV (n = 54)^a^
*Ease of understanding (HEART questions only)*					
Very easy	70%	NA	NA	NA	NA
Somewhat easy	20%	NA	NA	NA	NA
Somewhat difficult	10%	NA	NA	NA	NA
Very difficult	1%	NA	NA	NA	NA
*Relevance*					
Highly relevant	25%	54%	57%	67%	47%
Somewhat relevant	61%	39%	43%	26%	31%
Not very relevant	14%	7%	0%	7%	22%
*Helpfulness*					
Very helpful	79%	83%	90%	89%	73%
Somewhat helpful	18%	17%	10%	7%	20%
Not helpful	2%	0%	0%	3%	7%
*Length*					
Too long	25%	18%	13%	7%	11%
About right	61%	68%	83%	78%	74%
Not long enough	14%	13%	3%	15%	15%
*Preference for computer or counselor administration*					
Strongly prefer computer	36%	27%	27%	22%	30%
Somewhat prefer computer	2%	2%	3%	4%	4%
No preference	13%	16%	20%	19%	19%
Somewhat prefer a counselor	8%	4%	7%	0%	7%
Strongly prefer a counselor	41%	50%	43%	56%	41%

^a^All 92 participants received the healthy relationships counseling module and at least one other counseling module

Staff estimated that the HEART took 20–30 min on average to complete. Most participants (61%) thought that it took about the right amount of time, but 25% thought it took too long (Table 2). Participants had strong preferences for how they would like to answer the HEART questions in the future, with slightly more strongly preferring administration by a counselor (41%) than strongly preferring self-administration by computer (36%). For those who preferred a computer, reasons included that they would be able to answer the questions more honestly without being judged and they would have more control over the pacing (going faster, repeating questions, or going back to revise responses to earlier questions). As one respondent said, *“I feel that discussing my personal issues with a stranger is not okay, and with a computer I will be able to say everything that is personal without being shy”* (Siphokazi, age 25–29). For women who preferred a counselor, reasons included that they appreciated the sympathy and human touch of a counselor, and that a counselor could answer questions and provide advice. As one respondent said, *“Anything that you find hard to understand can be explained better by a counsellor than a computer”* (Thembekile, age 20–24).

For the healthy relationships, partner communication, and ring disclosure counseling modules, nearly all participants (93–100%) that received those modules thought they were highly or somewhat relevant (Table 2); fewer participants (78%) thought that the IPV module was relevant. A large majority (83–90%) said that the healthy relationships, partner communication, and ring disclosure modules were very helpful; a slightly smaller majority (73%) said that the IPV module was very helpful. In response to open-ended questions about the counseling modules, participants said that the modules increased their awareness of harmful dynamics in their relationships, improved their communication with their partners, and helped them talk to their partners about their ring use. Table 3 provides illustrative quotes to demonstrate participant reactions to each counseling module type. Most women who received the IPV counseling said that it empowered them, but some said that it was not relevant to them.

**Table 3:** Participant reactions to counseling modules

*Healthy Relationships Counseling*
I liked that I was able to talk to a stranger about my relationship because he or she will not be judgmental or take sides. (Zanele, age 30–34)
Talking to the counselor made me see things in a different angle, it made me realize that there are some things that I was doing towards my partner unaware that they are not right. (Nozizwe, age 20–24)
*Partner Communication Counseling*
I liked the module because it worked for me greatly. I was just a person who would keep quiet whenever I do not like something that my partner does. This module encouraged me to talk to my partner about what I don’t like…in a constructive manner. (Duduzile, age 35–39)
I liked that the counsellor touched on anger issues affecting communication in our relationship and how to calm down even when angry and talk things out instead of adding fuel in the fire. (Kholwa, age 25–29)
*Ring Disclosure Counseling*
I liked that they gave me ideas on ring disclosure and it worked—my partner now knows I’m using the ring and he doesn’t have a problem with it. (Mbalenhle, age 25–29)
*IPV Counseling*
It made me realize that I don’t have to let anyone control me and to stay in an abusive relationship (Sihle, age 20–24)
I did not like that the tool chose for me this module while I was not going through any abuse in my relationship. (Unathi, age 30–34)

Staff estimated that, on average, counseling at enrollment took 30–40 min. Most participants thought the counseling modules were about the right length (68–83%, depending on the module; Table 2). Nearly all participants (94% or more) rated the counselors as “great” or “good” in terms of their respect and caring, listening skills, confidentiality, and knowledge (not shown). Many more participants said that they would strongly prefer to receive the counseling modules in-person (41–56%) than said they would strongly prefer a hypothetical computer-based version of the counseling (22–30%). The reasons for their preferences were similar to the reasons they gave for completing the HEART with a counselor as compared with by themselves.

### Perceived impact of CHARISMA

**Fig. 1:**
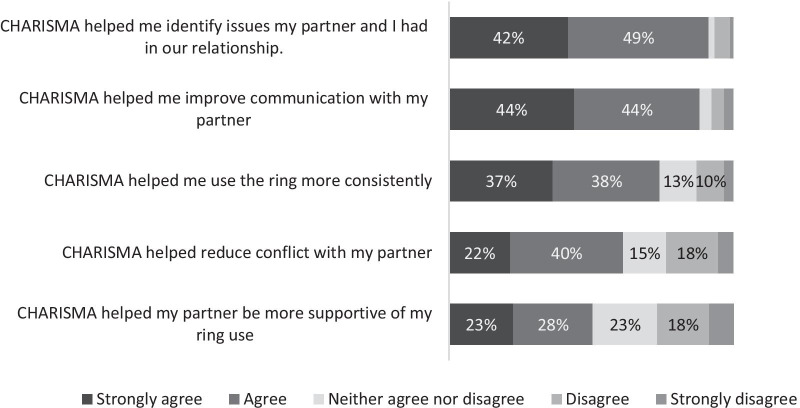
Participant perspectives on the impact of CHARISMA

A large majority of participants agreed or strongly agreed that CHARISMA had helped them to improve their relationships (91%) and to use the ring more consistently (88%; Fig. 1). Smaller majorities also agreed or strongly agreed that it had helped them use the ring more consistently (75%) and reduce conflict with their partners (62%), and that it helped their partner be more supportive of their ring use (51%).

In regard to ring disclosure, 35 participants (38%) said that when they enrolled in HOPE their partner did not know that they were using the ring or that they did not know if he knew (not shown). Of those 35, 12 (34%) reported that they had since told their partner that they were using the ring, and nine (75%) of them said that CHARISMA had been either very helpful (58.3%) or somewhat helpful (17%) in helping them talk to their partner about it (Table 4). Twenty-three participants had still not disclosed their ring use to their partner at the time of the survey, and 19 (83%) of this subset reported that CHARISMA had been either very helpful (65%) or somewhat helpful (17%) in helping them feel comfortable keeping their ring use a secret from their partner.

**Table 4:** Impact of CHARISMA on ring disclosure and IPV among select subgroups

Population subgroup	Measure	n (%)
Participants who disclosed ring use to partner after enrollment (n = 12)	Helpfulness of CHARISMA in talking to partner about ring use	
	Very helpful	7 (58)
	Somewhat helpful	2 (17)
	Made no difference	3 (25)
Participants who had not disclosed ring use to partner at time of interview (n = 23)	Helpfulness of CHARISMA in helping participant feel comfortable keeping ring use a secret from partner	
	Very helpful	15 (65)
	Somewhat helpful	4 (17)
	Made no difference	4 (17)
Participants whose partner was controlling or abusive at enrollment (n = 13)	Status of relationship at time of interview	
	No longer with that partner	3 (23)
	Still with that partner, but he is no longer controlling or abusive	7 (54)
	Still with that partner and he is still controlling or abusive, but have some strategies and information that make me feel safer than before	1 (8)
	Relationship has not changed	2 (15)
Participants whose relationship was controlling or abusive at enrollment and whose relationship status had changed at time of interview (n = 11)	How much of an effect CHARISMA had in bringing about that change in the relationship	
	A big effect	8 (73)
	A medium effect	2 (18)
	A small effect	0 (0)
	No effect	1 (9)

In regard to IPV, 13 participants (14%) said that their partner was controlling or abusive when they enrolled in HOPE and CHARISMA. Seven (54%) of these said that their partner was no longer controlling or abusive at the time of the survey (6 or more months after enrollment), and three (23%) said that they were no longer with that partner. One participant (8%) responded that her partner was still abusive, but she had some strategies and information that made her feel safer than before. Only 2 of the participants (15%) in abusive or controlling relationships at enrollment said that their relationship had not changed six months later. Of the 11 participants who said that their relationship had changed, eight (73%) said that CHARISMA had had a “big” effect in bringing about that change.

### Factors affecting the feasibility of charisma

#### Facilitators

Factors that facilitated CHARISMA implementation and effectiveness included the longitudinal design of CHARISMA, the use of lay counselors, and implementation of CHARISMA at Wits RHI as part of the existing HOPE study.

Staff thought that the fact that CHARISMA had multiple visits over time was important in enhancing rapport between clinic staff and participants, which could increase the effectiveness of the intervention. Staff observed that some participants were reluctant to open up about difficulties in their relationships during the enrollment visit but they believed that participants became more comfortable over time, and more open or honest in subsequent visits. Also, staff said the ongoing support is important because, from their perspective, IPV is so normalized in South Africa that participants need time to reach a stage where they recognize IPV in their relationship and are ready to take action on it.

As noted previously, one way in which CHARISMA differed from the Safe & Sound intervention was that CHARISMA used lay counselors instead of nurses to administer the intervention. Clinic supervisors thought that the lay counselors were perceived by study participants as relatable and attentive, caring people (a perspective that was confirmed by the participant survey results). Supervisors also noted that use of lay counselors facilitated hiring, because many people in Johannesburg are certified HIV counselors, a key qualification for the position, and a large pool of candidates had basic, related skills and training. Finally, supervisors commented that lay counselors are a lower wage category of staff to hire than nurses, which reduces the overall cost of the intervention and could make the approach more feasible to fund and implement in public health clinics in the future.

Staff believed that implementation of CHARISMA at the Wits RHI clinic as part of the HOPE study facilitated implementation in several ways. Because nearly all of the CHARISMA staff had worked on ASPIRE, the clinical trial of the dapivirine ring that preceded HOPE, they had seen the need for an intervention like CHARISMA, which added to their motivation and commitment to the work. Further, counseling staff were hired solely to work on CHARISMA and to offer HIV pre- and post-test counseling; as a result, they did not have multiple competing work priorities, as might be the case in a public health clinic. Finally, staff said that the quality of care in the Wits RHI clinic was very high and very personal, and because participants had been coming to the clinic for years (since enrollment in the ASPIRE trial, which began in November 2012), they felt comfortable there and trusted in the confidentiality of the services.

#### Challenges

Challenges to implementation included the length of time required to administer CHARISMA, counselor stress from hearing participants’ traumatic stories (vicarious trauma), limited male partner engagement, participants’ lack of follow-through on referrals, and external contextual factors.

The staff noted that the CHARISMA intervention was lengthy to administer. From start to finish (including time for the participant to enter the clinic and sign in, introductions, overview of CHARISMA, informed consent, administration of the HEART, provision of counseling, discussion of referrals, and signing out), staff estimated that the intervention typically took 1.5–2 h at the enrollment visit, and 45 min to an hour for the month 1 visit (including sign-in, follow-up counseling, discussion of referrals, and sign-out). In the absence of further intervention streamlining, this could present an important barrier to successfully implementing the intervention in a public health clinic.

Staff commented that hearing difficult stories about participants’ experiences with IPV was stressful for the counselors. To address the stress, clinic supervisors were available to any of the counselors who needed to talk, and the team met regularly to process the issues they were dealing with and support each other. The counselors felt that this helped them regulate their emotions, build skills, and feel more cohesive as a team. Counselors were also able to take a break after any counseling sessions that were particularly stressful. Counselors thought that these measures were insufficient, however, to enable them to fully deal with the stress and symptoms of vicarious trauma. To provide additional support, the project hired a psychologist, who began holding bi-monthly group debriefing sessions with the team and was also available for individual counseling sessions when needed. At the time of the KIIs, the psychologist had been assisting for only two weeks, but the staff perceived her as helpful.

Engaging participants’ male partners was challenging. By Month 6, male partners of just 14 participants (15%) had come to the clinic. Forty-four percent of participants in the survey said they had not invited their partners to come to the clinic. The community outreach component of CHARISMA reached over 10,000 men, but none of the women participating in CHARISMA said that their partners had participated in any of these activities. Efforts to invite the participants’ male partners to the community outreach events were hampered by the need to preserve the confidentiality of the participants and by challenges in coordination between the organizations responsible for the clinic and community components of CHARISMA.

Relatively few participants followed through on referrals from clinic staff to external organizations. Twenty-nine participants (32%) reported that they had received a referral for services outside the clinic, and 10 (35% of those who received a referral) said that they had gone for the services. The primary reasons given for not following through on a referral were that they did not think they had a problem, the problem had been resolved, they knew how to resolve the problem, or they did not have time. Staff surmised that participants may be willing to tell their story or receive services and care in the research clinic, which is a familiar place, but may not be willing to do so at an unfamiliar place. In addition, staff thought that in cases where participants’ problems involved their partners, they might feel like they were betraying their partner if they followed through on the referral. The staff tried to help overcome the barriers by offering transportation and accompaniment to the referral organizations, but this was often insufficient to overcome the participants’ reluctance to seek external organizations’ assistance.

Several external contextual factors posed challenges to the successful implementation of CHARISMA. As previously mentioned, staff commented that IPV is so common that it is normalized in Johannesburg, so helping women (and their partners) see it as a problem, or as addressable, can take time. Staff also noted that people of some cultures in South Africa do not talk about their problems, so some participants may have been reluctant to speak openly with counselors. Staff believed that the intensive nature of the intervention, with more than one session, was key to overcoming these barriers and making intervention impact feasible. Finally, staff commented that another challenge was the lack of formal commitment in many of the women’s relationships (only 17% were married, and 34% were cohabiting), which may have made male partners less willing to work to improve the relationship and limited the women’s ability to insist that they do so.

## Discussion

The CHARISMA pilot study indicates that a two-session relationship counseling intervention with 6-month follow-up to support women’s ability to safely and effectively use vaginal microbicides was generally acceptable and feasible. Based on these promising preliminary findings, the project team is currently conducting a randomized controlled trial (RCT) in which participants receive either the standard of care for IPV screening and referral (control arm) or the CHARISMA intervention. The RCT will rigorously measure the effectiveness of CHARISMA at increasing oral PrEP adherence, partner communication, and partner support, and at decreasing IPV and social harms. For the RCT, we have made some minor modifications to the intervention in response to the participants’ feedback. For example, since a quarter of participants who received the IPV module said that it was not very relevant to them because their partner was not abusive or controlling, we adjusted the HEART scoring algorithm so that it is less likely to recommend IPV counseling for participants who have borderline indications of control or abuse in their relationships and updated the IPV counseling module to discuss different types of violence and controlling behaviors to help women recognize these dynamics in relationships. In addition, because oral PrEP is currently being scaled up in South Africa and the dapivirine vaginal ring is undergoing regulatory review, the CHARISMA RCT is offering oral PrEP rather than the vaginal ring; we therefore modified the HEART and counseling materials to reflect this change. Finally, community outreach activities have been replaced with clinic invitation letters and more targeted informational materials for participant’s partners.

The pilot study offers several lessons learned regarding the implementation of CHARISMA that may be applicable to similar interventions. Clinic staff felt strongly that the length and intensity of CHARISMA (core session, booster session, 6-month follow-up period, and repetition of the intervention for any women with new partners) were necessary for participants to begin to trust the counselors enough to be open and honest about problems in their relationships and to begin to overcome the normalization of IPV, and most participants thought that CHARISMA’s length was about right. Its length and intensity may not be feasible in some settings, but, based on the staff and participant feedback, a shorter, less-intensive version of the intervention may not offer as much support as women need. The value of having a longer intervention is further supported by the fact that most IPV interventions involve multiple sessions over a period of weeks or months [24]. One possible approach to reduce the burden on clinic staff would be to have some or all components of the intervention self-administered through a computer rather than requiring a counselor [25–29]. Between a quarter and a third of participants reported that they would strongly prefer to have CHARISMA self-implemented on a computer, so this could be a good option for at least some women. However, more women (40–56%) said that they would strongly prefer to have it administered by a counselor. Ideally, if testing shows that both the counselor and computer-based versions of CHARISMA are effective, it could be made available to women in both formats or a mixture of the two.

Because male partner awareness and support is likely to play an important role in women’s ability to successfully use an HIV prevention product [4–6], interventions should target both men and women. However, similar to previous studies of vaginal microbicides, engaging women’s male partners in CHARISMA was challenging [5]. Most women did not invite their partners to the clinic, and very few of the invited partners came to the clinic. The community-level outreach to men succeeded in educating a large number of men in the community, but we have no evidence that any of the men it reached were partners of the women in the study. To try to reach more male partners for the CHARISMA RCT, clinic staff are offering to call participants’ partners to talk to them directly about the study and/or PrEP and giving participants a packet of materials for their male partners that includes educational materials on HIV and STI prevention and treatment, PrEP, and gender issues and violence. These measures may help to educate participants’ male partners about the study and issues related to HIV prevention without the need for them to come to the clinic, and to help address any common male suspicions about HIV prevention product use at product use initiation. The packet also includes a letter that invites the male partner to come to the clinic for individual or couples counseling on partner communication, STI and HIV prevention in their relationship and any other topics they may need help on, and offers free HIV testing and STI counseling and treatment.

The use of lay counselors instead of nurses appeared to be successful. Participants gave the counselors very high ratings, and lay counselors are less expensive and easier to hire than nurses. Previous research supports the effectiveness of lay counselors instead of more formally trained health professionals [30–32]. However, counselors did experience significant stress hearing about the traumas experienced by some participants, and providing adequate emotional support was important to staff well-being. This finding is similar to those of previous studies documenting the risk of vicarious trauma and job burnout among mental health workers working with traumatized clients, including among HIV counselors in South Africa [33–36]. The level of support required to help counselors manage the stress could be challenging to provide in the context of the public health system.

This study is subject to several limitations. Social desirability bias may have led participants to rate CHARISMA overall, CHARISMA counseling, or in-person as compared with computerized counseling more positively than they actually felt, especially because questionnaires were administered in the Wits RHI clinic by colleagues of CHARISMA counselors. Social desirability bias may also have led CHARISMA staff to downplay any problems related to CHARISMA and its implementation, although staff did raise several challenges. Embedding CHARISMA within the context of the HOPE study at the Wits RHI clinic was a unique context, and issues related to the feasibility and acceptability of the intervention may differ in other settings. Similarly, because participants were not naïve users of the dapivirine ring, their experiences may differ from women using an HIV prevention product for the first time. To address some of these limitations, the RCT will be conducted with naïve users of oral PrEP, using a randomized design.

## Conclusions

In conclusion, enhancing male partner support and mitigating the potential for intimate partner violence or other social harms related to women’s use of HIV prevention products is critical to empowering women to protect themselves from HIV. The pilot study results suggest that the CHARISMA counseling intervention is a feasible and acceptable approach to this end. The RCT currently underway will provide more definitive results regarding the CHARISMA’s effectiveness at increasing HIV prevention product adherence, disclosure and partner support and decreasing IPV and social harms. Lessons learned from the pilot that are relevant to other interventions include the desirability of options for either in-person or computer-based counseling, depending on participant preferences; challenges related to male partner engagement, both through clinic outreach and community-level outreach; and the successful use of lay counselors rather than health professionals to implement the counseling. Staff perceptions about the perceived benefit of providing a longitudinal intervention over a period of several months may also be relevant to similar interventions, although additional research is needed to determine an intervention delivery and follow-up period that optimally balances participant needs and clinic resources.

## Supplementary Information

**Additional file 1.** Survey questionnaire.

## Data Availability

The datasets generated and/or analyzed during the current study are not publicly available due concerns about participant privacy but are available from the corresponding author on reasonable request and with use of a data use agreement to protect participant privacy.

## Abbreviations

ASPIRE: A Study to Prevent Infection with a Ring for Extended Use
CFIR: Consolidated Framework for Implementation Research
CHARISMA: Community Health Clinic Model for Agency in Relationships and Safer Microbicide Adherence
HEART: HEAlthy Relationship assessment Tool
HOPE: HIV Open-Label Prevention Extension
IPV: Interpersonal violence
IRB: Institutional Review Board
KII: Key informant interviews
PrEP: Pre-exposure prophylaxis
RCT: Randomized controlled trial
STI: Sexually transmitted infection
Wits RHI: Wits Reproductive Health and HIV Institute
