# Supplementary material for: Acceptability and feasibility of the CHARISMA counseling intervention to support women’s use of pre-exposure prophylaxis: results of a pilot study
Source: BMC Womens Health. 2021 Mar 25;21:126. doi: 10.1186/s12905-021-01262-z (PMC7992829; doi:10.1186/s12905-021-01262-z)
Supplement: Supplementary file 1 — Additional file 1. Survey questionnaire. [file 12905_2021_1262_MOESM1_ESM.docx]

| ***Instructions:*** *Pre-populate the background question responses below prior to meeting with each participant. For sections following this one, read each question word for word to the participant. Unless otherwise indicated, only one response may be selected. Italicized text should not be read to participants.* | | |
| --- | --- | --- |
| **Background information** | | |
|  | Which counseling modules has the participant received? (*Select all that apply.)* | _1_ Healthy relationships  _2_ Module A: Partner communication  _3_ Module A Component: Conflict de-escalation and resolution skills  _4_ Module B: Ring disclosure decisions and skills  _5_ Module C: Responding to intimate partner violence (IPV) |

| **Reactions to SBHT** | | |
| --- | --- | --- |
| At the start of CHARISMA and at some other follow-up visits, you were asked a series of questions that made up what we called the Social Benefits Harm Tool or SBHT. To refresh your memory, the questions were about men’s and women’s roles, your relationship with your partner, and you and your partner’s attitudes towards HIV prevention. (*Show a list of the SBHT questions)*. The next few questions we want to ask you are about the SBHT. Please think about the first time you were asked the SBHT questions. | | |
|  | Overall, how clear were the SBHT questions? By this I mean how well you understood the questions. *(Read response options)* | _1_ Very clear  _2_ Somewhat clear  _3_ Not clear at all  _4_ Don’t know/don’t remember |
|  | Thinking about the first time you were asked the SBHT questions, did you find it easy or difficult to be open and honest in answering the questions? *(Read response options)* | _1_ Very easy  _2_ Somewhat easy  _3_ Somewhat difficult  _4_ Very difficult  _5_ Don’t know/don’t remember |
|  | Did the SBHT make you feel emotional? By emotional we mean did it trigger any change in your feelings *(Read response options)* | _1_ Yes, very emotional  _2_ Yes, somewhat emotional  _3_  No, not at all emotional 🡪 Skip to Q5  _4_ Don’t know/don’t remember 🡪 Skip to Q5 |
|  | Could you please tell me what made you feel emotional? (You could tell us about both good and bad emotions) | (Free text): ______________________________________  ________________________________________________________________________________________________________________________________________________________________________________________________________________________________________________ |
|  | Would you say that the SBHT took too long, took about the right amount of time, or was not long enough? | _1_ Too long  _2_ About right amount of time  _3_ Not long enough |
|  | Thinking about the first time you were asked the SBHT questions, how helpful were they? *(Read response options)* | _1_ Very helpful  _2_ Somewhat helpful  _3_ Not helpful |
|  | Please explain why you found the SBHT helpful or unhelpful. | (Free text): ______________________________________  _______________________________________________________________________________________________________________________________________________________________________________________________________________________________________ |
|  | Could you please tell me which responses match your feelings about answering the SBHT questions? Please respond yes to each that matches your thoughts on the SBHT. *(Read response options and select all that apply)* | _1_ They helped me see ways my relationship could improve.  _2_ They made me feel encouraged to make changes in my relationship.  _3_ They made me feel guilty for not making changes to my relationship.  _4_ They made me feel sad or unhappy.  _5_ They were annoying.  _6_ It made me realize how much anger I have about my  relationship  _7_ I don’t have strong feelings about them. |
|  | When you were asked the SBHT questions at later study visits, do you think your responses changed from the first time you answered them? | _1_ Yes  _2_ No 🡪 Go to #11 |
|  | Why do you think your SBHT responses changed?  *(Read response options and select all that apply)* | _1_ My thinking changed  _2_ My circumstances changed  _3_ I understood the questions better when I heard them again  _4_ I felt more comfortable being open  _5_ I did not want to admit some of my problems because I didn’t feel like talking about them  _6_ Other (*specify)*______________________________  ______________________________________________ |
|  | In future would you prefer to answer the SBHT questions on a tablet or computer that you can read or listen to on a headset, or would you prefer to have a counselor ask you the questions? Do you…  *(Read response options)* | _1_ Strongly prefer computer  _2_ Somewhat prefer computer  _3_ Somewhat prefer a counselor  _4_ Strongly prefer a counselor  _5_ Have no preference |
|  | Please explain your thinking about your preference. *(insert response)* | (Free text):_______________________________________  ________________________________________________________________________________________________________________________________________________________________________________________________ |
|  | We are thinking about how to provide CHARISMA services in a wider range of locations, such as public health clinics. Would you be willing to answer the SBHT questions in a public clinic? If willing or unwilling please provide reasons. | (Free text):_______________________________________  ________________________________________________________________________________________________________________________________________________________________________________________________ |

| **Counseling modules** | | |
| --- | --- | --- |
| ***Instructions:*** *Based on the response to* *Background information Q1, determine which modules the participant has received, and ask the questions relevant to those modules only.* | | |
| Now we are going to ask you some questions about the counseling modules you received as part of CHARISMA. | | |
| **Healthy Relationships** | | |
| First I want to ask you about the Healthy Relationships counseling. The Healthy Relationships counseling is about what men and women want from relationships, and the types of abuse that may occur in unhealthy relationships. These types of abuse, as shown in the Wheel of Power, include emotional and psychological abuse, physical and sexual abuse, and financial abuse. The counselor then discussed your relationship, and the ways in which it may have been healthy or unhealthy. (*Show participant the pages in the flip chart for the Healthy Relationships section as you describe its content to remind her of what was in it)*. | | |
|  | How relevant to you were the issues covered in the healthy relationships counseling? *(Read response options)* | _1_ Highly relevant  _2_ Somewhat relevant  _3_ Not very relevant |
|  | How helpful was the information presented in this counseling section? *(Read response options)* | _1_ Very helpful  _2_ Somewhat helpful  _3_ Not helpful |
|  | Would you say that the healthy relationships counseling took too long, took about the right amount of time, or was not long enough? | _1_ Too long  _2_ About right amount of time  _3_ Not long enough |
|  | Is there anything else you would like to say about what you liked or disliked about the healthy relationships counseling? | (Free text):_____________________________  ___________________________________________________________________________________________________________________________________________________________________________________________________ |
|  | How would you feel about having the information in the healthy relationships counseling presented on a tablet or computer with audio or having it presented by a counsellor? Do you…*(Read response options)* | _1_ Strongly prefer computer  _2_ Somewhat prefer computer  _3_ Somewhat prefer counsellor  _4_ Strongly prefer counsellor  _5_ Have no preference 🡪 Skip to the next module |
|  | Please explain your thinking about your preference of a tablet or computer with audio or having it presented in-person by a counsellor. *(insert response)* | (Free text):_____________________________  ___________________________________________________________________________________________________________________________________________________________________________________________________ |
|  | | |

| **Partner Communication** | | |
| --- | --- | --- |
| Now I want to ask you about the Partner Communication counseling you received. The Partner Communication counseling talks about how words, voice and tone, and body language affect communication. It also talks about using “I” statements to deal with conflict. (*Show participant the pages in the flip chart for the Partner Communication section as you describe its content to remind her of what was in it)*. | | |
|  | How relevant to you were the issues covered in the partner communication counseling? *(Read response options)* | _1_ Highly relevant  _2_ Somewhat relevant  _3_ Not very relevant |
|  | How helpful was the information presented in this counseling section? *(Read response options)* | _1_ Very helpful  _2_ Somewhat helpful  _3_ Not helpful |
|  | Since learning about “I” statements during counseling, how many times have you used an “I” statement in an interaction with your partner or another person? Would you say you… *(Read response options)* | _1_ Have not used “I” statements yet  _2_ Have used them in 1-3 interactions  _3_ Have used them in 4-6 interactions  4 Have used them in 7 or more interactions |
|  | Would you say that the partner communication counseling took too long, took about the right amount of time, or was not long enough? | _1_ Too long  _2_ About right amount of time  _3_ Not long enough |
|  | Is there anything else you would like to say about what you liked or disliked about partner communication counseling? | (Free text):_____________________________  ___________________________________________________________________________________________________________________________________________________________________________________________________ |
|  | How would you feel about having the information in partner communication counseling presented on a tablet or computer with audio or having it presented in-person by a counselor? Do you…*(Read response options)* | _1_ Strongly prefer computer  _2_ Somewhat prefer computer  _3_ Somewhat prefer counsellor  _4_ Strongly prefer counsellor  _5_ Have no preference |
|  | Please explain your thinking about your preference of a tablet or computer with audio or having it presented in-person by a counselor. *(insert response)* | (Free text):_____________________________  ___________________________________________________________________________________________________________________________________________________________________________________________________ |

| **Conflict De-Escalation and Resolution Skills** | | |
| --- | --- | --- |
| Now I want to ask you about the Conflict De-Escalation and Resolution counseling you received. This counseling talks about how to avoid “unfair or dirty fighting” when you have a conflict with your partner, and to stay focused on the issue instead of escalating to the personality level or the relationship level. (*Show participant the pages in the flip chart for the Conflict Resolution section as you describe its content to remind her of what was in it)*. | | |
|  | How relevant to you were the issues covered in the conflict resolution counseling? Would you say that it was… *(Read response options)* | _1_ Highly relevant  _2_ Somewhat relevant  _3_ Not very relevant |
|  | How helpful was the information presented in this counseling section? Would you say that it was… *(Read response options)* | _1_ Very helpful  _2_ Somewhat helpful  _3_ Not helpful |
|  | Would you say that the conflict resolution counseling took too long, took about the right amount of time, or was not long enough? | _1_ Too long  _2_ About right amount of time  _3_ Not long enough |
|  | Is there anything else you would like to say about what you liked or disliked about conflict resolution counseling? | (Free text):_____________________________  ___________________________________________________________________________________________________________________________________________________________________________________________________ |
|  | How would you feel about having the information in conflict resolution counseling presented on a tablet or computer with audio or having it presented in-person by a counselor? Do you…*(Read response options)* | _1_ Strongly prefer computer  _2_ Somewhat prefer computer  _3_ Somewhat prefer counsellor  _4_ Strongly prefer counsellor  _5_ Have no preference |
|  | Please explain your thinking about your preference of a tablet or computer with audio or having it presented in-person by a counselor. *(insert response)* | (Free text):_____________________________  ___________________________________________________________________________________________________________________________________________________________________________________________________ |

| **Ring Disclosure Decisions and Skills** | | |
| --- | --- | --- |
| Now I want to ask you about the ring disclosure counseling you received. This counseling talked about reasons why you might or might not want to tell your partner that you are using the ring, what kinds of reactions a man might have to hearing his partner was using the ring, and how a woman might respond. If you decided you wanted to tell your partner about the ring, it covered how best to do that, and if you decided you did not want to tell him, it covered ideas for how to keep him from finding out. (*Show participant the pages in the flip chart for the ring disclosure section as you describe its content to remind her of what was in it)*. | | |
|  | How relevant to you were the issues covered in the ring disclosure counseling? Would you say that it was… *(Read response options)* | _1_ Highly relevant  _2_ Somewhat relevant  _3_ Not very relevant |
|  | How helpful was the information presented in this counseling section? Would you say that it was… *(Read response options)* | _1_ Very helpful  _2_ Somewhat helpful  _3_ Not helpful |
|  | Would you say that the ring disclosure counseling took too long, took about the right amount of time, or was not long enough? | _1_ Too long  _2_ About right amount of time  _3_ Not long enough |
|  | Is there anything else you would like to say about what you liked or disliked about ring disclosure counseling? | (Free text):_____________________________  ___________________________________________________________________________________________________________________________________________________________________________________________________ |
|  | How would you feel about having the information in ring disclosure counseling presented on a tablet or computer with audio or having it presented in-person by a counselor? Do you…*(Read response options)* | _1_ Strongly prefer computer  _2_ Somewhat prefer computer  _3_ Somewhat prefer counsellor  _4_ Strongly prefer counsellor  _5_ Have no preference |
|  | Please explain your thinking about your preference of a tablet or computer with audio or having it presented in-person by a counselor. *(insert response)* | (Free text):_____________________________  ___________________________________________________________________________________________________________________________________________________________________________________________________ |

| **IPV** | | |
| --- | --- | --- |
| Now I want to ask you about the Intimate Partner Violence counseling you received. This counseling covered the “cycle of violence,” where there is a calm phase, and then a phase where tension builds, until the tension is broken by a violent episode. It also talked about creating a safety plan. (*Show participant the pages in the flip chart for the IPV section as you describe its content to remind her of what was in it)*. | | |
|  | How relevant to you were the issues covered in the IPV counseling? Would you say that it was… *(Read response options)* | _1_ Highly relevant  _2_ Somewhat relevant  _3_ Not very relevant |
|  | How helpful was the information presented in this counseling section? Would you say that it was… *(Read response options)* | _1_ Very helpful  _2_ Somewhat helpful  _3_ Not helpful |
|  | Would you say that the IPV counseling took too long, took about the right amount of time, or was not long enough? *(Read response options)* | _1_ Too long  _2_ About right amount of time  _3_ Not long enough |
|  | Is there anything else you would like to say about what you liked or disliked about IPV counseling? | (Free text):_____________________________  ___________________________________________________________________________________________________________________________________________________________________________________________________ |
|  | How would you feel about having the information in IPV counseling presented on a tablet or computer with audio or having it presented in-person by a counselor? Do you…*(Read response options)* | _1_ Strongly prefer computer  _2_ Somewhat prefer computer  _3_ Somewhat prefer counsellor  _4_ Strongly prefer counsellor  _5_ Have no preference |
|  | Please explain your thinking about your preference of a tablet or computer with audio or having it presented in-person by a counselor. *(insert response)* | (Free text):_____________________________  ___________________________________________________________________________________________________________________________________________________________________________________________________ |

| **Overall visit** | |
| --- | --- |
|  | On average, please rate the CHARISMA counselor(s) you spoke with in the following areas.  *(Show participant Showcard A with response options)*   \|  \| Great \| Good \| Average \| Poor \| Very Poor \| \| --- \| --- \| --- \| --- \| --- \| --- \| \| 1. Respect/caring \| \| _1_ \| _2_ \| _3_ \| _4_ \| _5_ \| \| 1. Listening skills (good listener) \| \| _1_ \| _2_ \| _3_ \| _4_ \| _5_ \| \| 1. Confidentiality \| \| _1_ \| _2_ \| _3_ \| _4_ \| _5_ \| \| 1. Knowledge \| \| _1_ \| _2_ \| _3_ \| _4_ \| _5_ \| |
|  | Please rate your overall satisfaction with the CHARISMA counselor or counselors.  *(Show participant Showcard A with response options)*   \|  \| Great \| Good \| Average \| Poor \| Very Poor \| \| --- \| --- \| --- \| --- \| --- \| --- \| \| 1. Overall satisfaction \| \| _1_ \| _2_ \| _3_ \| _4_ \| _5_ \| |

|  | \| Please tell us how much you agree or disagree with the following statements.  *(Show participant Showcard B with response options)* \| Strongly Disagree \| Disagree \| Neither Agree nor Disagree \| Agree \| Strongly Agree \| \| --- \| --- \| --- \| --- \| --- \| --- \| \| 1. CHARISMA helped me identify issues my partner and I had in our relationship. \| _1_ \| _2_ \| _3_ \| _4_ \| _5_ \| \| 1. CHARISMA helped me improve communication with my partner. \| _1_ \| _2_ \| _3_ \| _4_ \| _5_ \| \| 1. CHARISMA helped my partner be more supportive of my ring use. \| _1_ \| _2_ \| _3_ \| _4_ \| _5_ \| \| 1. CHARISMA helped reduce conflict with my partner. \| _1_ \| _2_ \| _3_ \| _4_ \| _5_ \| \| 1. CHARISMA helped me use the ring more consistently. \| _1_ \| _2_ \| _3_ \| _4_ \| _5_ \| \| 1. CHARISMA made me feel more confident. \| _1_ \| _2_ \| _3_ \| _4_ \| _5_ \| \| 1. CHARISMA made me feel sad or unhappy. \| _1_ \| _2_ \| _3_ \| _4_ \| _5_ \| \| 1. CHARISMA made me feel guilty for not making changes to my relationship. \| _1_ \| _2_ \| _3_ \| _4_ \| _5_ \| \| 1. CHARISMA made me feel pressured to make changes in my relationship I was not ready for. \| _1_ \| _2_ \| _3_ \| _4_ \| _5_ \| \| 1. The counselors supported me to make my own decisions about what was right for me \| _1_ \| _2_ \| _3_ \| _4_ \| _5_ \| |
| --- | --- | --- | --- | --- | --- | --- | --- | --- | --- | --- | --- | --- | --- | --- | --- | --- | --- | --- | --- | --- | --- | --- | --- | --- | --- | --- | --- | --- | --- | --- | --- | --- | --- | --- | --- | --- | --- | --- | --- | --- | --- | --- | --- | --- | --- | --- | --- | --- | --- | --- | --- | --- | --- | --- | --- | --- | --- | --- | --- | --- | --- | --- | --- | --- | --- | --- | --- |

|  | At enrollment in HOPE, did your partner know that you might be using the ring? | _1_ Yes 🡪 Skip to question #8  _2_ No  _3_ Don’t know |
| --- | --- | --- |
|  | Have you told your partner since then that you are using it? | _1_ Yes  _2_ No 🡪 Skip to question #7  _3_ NA, I am not using the ring 🡪 Skip to question #7 |
|  | How helpful would you say CHARISMA was in helping you talk to your partner about your use of the ring? *(Read response options)* | _1_ Very helpful 🡪 Skip to question #8  _2_ Somewhat helpful 🡪 Skip to question #8  _3_ Made no difference 🡪 Skip to question #8 |
|  | How helpful would you say CHARISMA was in helping you feel comfortable keeping your ring use a secret from your partner? *(Read response options)* | _1_ Very helpful  _2_ Somewhat helpful  _3_ Made no difference |
|  | At enrollment in HOPE, would you say that your partner was controlling or abusive? | _1_ Yes  _2_ No 🡪 Skip to question #11 |
|  | Which of the following best describes your relationship with that partner now? *(Read response options)* | _1_ I am no longer with that partner  _2_ I am still with that partner, but he is no longer controlling or abusive  _3_ I am still with that partner and he is still controlling or abusive, but I have some strategies and information that make me feel safer than before  _4_ My relationship with my partner has not changed. 🡪 Skip to question #11  _5_ Other, specify: ____________________________________ |
|  | How much of an effect would you say that CHARISMA had in bringing about this change in your relationship? Would you say that it had… *(Read response options)* | _1_ A big effect  _2_ A medium effect  _3_ A small effect  _4_ No effect |
|  | Have you shared information you learned from CHARISMA counseling or referrals with anyone? | _1_ Yes (specify who, e.g., friend, sister, neighbor)  Specify: _________________________________  _2_ No |
|  | Would you recommend CHARISMA to a friend? Would you say… *(Read response options)* | _1_ Definitely  _2_ Probably  _3_ Probably not  _4_ Definitely not |
|  | Did you ever receive a referral for services outside the Wits RHI clinic? | _1_ Yes  _2_ No 🡪 Go to #16 |
|  | Did you go to the place you were referred to? | _1_ Yes 🡪 Go to #16  _2_ No |
|  | Why not? | (Free text): ____________________________________________________________________________________________________________________________________________________________ |
|  | Did you ever invite your partner to come to the clinic? | _1_ Yes 🡪 Go to #18  _2_ No |
|  | Why not? | (Free text): ____________________________________________________________________________________________________________________________________________________________ |
|  | Did you ever receive information on Whatsapp about events for men? | _1_ Yes  _2_ No 🡪 Go to #21 |
|  | Did you ever share the event information with your partner? | _1_ Yes 🡪 Go to #21  _2_ No |
|  | Why not? | (Free text): ____________________________________________________________________________________________________________________________________________________________ |
|  | Is there anything else you want to share with me about what you liked or disliked about CHARISMA? | (Free text): ____________________________________________________________________________________________________________________________________________________________________________________________________________________________________________________________________________________________________________________________________________________________________________ |
|  | Do you have any suggestions on what we can do to improve CHARISMA? | (Free text): ____________________________________________________________________________________________________________________________________________________________________________________________________________________________________________________________________________________________________________________________________________________________________________ |
